# Comparison of Neck Muscle Thickness via Ultrasound in Patients with Ankylosing Spondylitis and Healthy Controls

**DOI:** 10.5152/ArchRheumatol.2026.25253

**Published:** 2026-04-24

**Authors:** Derya Karacif, Onur Karacif, Ayla Cagliyan Turk

**Affiliations:** 1 Department of Physical Medicine and Rehabilitation, Erol Olçok Training and Research Hospital, Çorum, Türkiye; 2 Department of Radiology, Erol Olçok Training and Research Hospital, Çorum, Türkiye; 3 Department of Physical Medicine and Rehabilitation, Hitit University Faculty of Medicine, Çorum, Türkiye

**Keywords:** Ankylosing spondylitis, neck muscles, quality of life, ultrasound

## Abstract

**Background/Aims:**

: Neck pain and limited neck mobility are common symptoms in patients with ankylosing spondylitis (AS). This study aimed to quantify neck muscle thickness in individuals with AS using ultrasonography and to compare these findings with measurements obtained from healthy volunteers.

**Materials and Methods:**

A total of 30 individuals diagnosed with AS and 30 healthy participants were enrolled in this case–control study. The anteroposterior dimension, lateral dimension, cross-sectional area (CSA) of the longus colli (LC), cervical multifidus (CM), and anteroposterior dimension of the sternocleidomastoid were measured via ultrasound. Individuals diagnosed with AS were asked to complete the Neck Disability Index (NDI), the Ankylosing Spondylitis Quality of Life questionnaire (ASQoL), the Bath Ankylosing Spondylitis Activity Index (BASDAI), the Bath Ankylosing Spondylitis Functional Index (BASFI), and the Bath Ankylosing Spondylitis Metrology Index (BASMI).

**Results:**

In the AS group, the anteroposterior thickness (*P* = .025) and CSA (*P* = .021) of the right LC were markedly reduced compared with the control group. Similarly, the left LC exhibited significantly smaller anteroposterior measurements (*P* = .023) and CSA values (*P* = .012) in patients with AS. Additionally, the lateral dimension of the CM was significantly decreased on both the right (*P* = .001) and left (*P* = .002) sides relative to healthy controls. In patients with AS, LC thickness correlated negatively with NDI, ASQoL, BASDAI, BASFI, and BASMI, and positively with cervical extension and lateral flexion. In multivariate regression analyses adjusted for age and body mass index, LC thickness was independently associated with disability, functional impairment, and spinal mobility limitation.

**Conclusion:**

: Neck muscle thickness is reduced in AS patients, and atrophy of the LC shows significant correlations with greater disability, poorer quality of life, higher disease activity, impaired functional status, and limited spinal mobility.

**Main Points**

- Neck muscle thickness is significantly reduced in patients with ankylosing spondylitis (AS) compared to healthy individuals.
- Longus colli (LC) atrophy shows significant correlations with disability, disease activity, quality of life, functional status, and spinal mobility.
- Reduced LC thickness is independently associated with greater disability, impaired functional status, and restricted spinal mobility.
- Ultrasound is an important tool in the evaluation of neck muscles in patients with AS.

## Introduction

Ankylosing spondylitis (AS) is a chronic, progressive rheumatic disease that affects mainly the axial skeleton and sacroiliac joints.^1^ In patients with AS, all the spinal segments, including the cervical spine, may be involved.^2^ In AS, the vertebral bodies, intervertebral discs, apophyseal joints, ligaments, and muscles adjacent to the spine may also be affected.

Neck pain and stiffness are common symptoms in patients with AS. Neck musculature plays an important role in stabilizing the cervical vertebrae, and dysfunction in these muscles may be closely associated with neck pain.^3^ In a study evaluating paravertebral muscle biopsies, atrophy of type-2 fibers was found in AS patients.^4^ A histopathological study revealed increased collagen fibril deposition and atrophy in the paraspinal muscle tissue of AS patients.^5^

The cervical multifidus (CM) is a key muscle contributing to cervical spine biomechanics and segmental stability.^6^ The morphology and histology of CM have been associated with various conditions such as chronic cervical radiculopathy,^7^ chronic idiopathic neck pain,^8^ and whiplash-related disorders.^9^ A study conducted in individuals with reduced cervical lordosis reported a notable decline in the strength of the cervical extensor muscles.^10^ As AS is associated with loss of cervical lordosis,^11^ neck extensor muscle weakness may occur in this population. The longus colli (LC) is one of the primary deep cervical flexor muscles, and previous research has demonstrated that individuals with chronic mechanical neck pain exhibit reduced LC thickness compared with healthy subjects.^12^ Individuals experiencing chronic radicular neck pain have also been shown to display atrophy of the LC muscle.^3^ The sternocleidomastoid (SCM) muscle is a superficial neck flexor muscle. Weakness or altered muscle activity of the SCM muscle has been reported to be associated with neck pain.^13^ Reduced neuromuscular activation of the SCM and anterior scalene muscles has been reported in individuals suffering from chronic neck pain.^14^ Falla and colleagues observed that patients with unilateral chronic neck pain exhibited fatigue of the superficial flexor muscles on the symptomatic side.^15^

Ultrasound has shown high reliability for the evaluation of neck muscles, supporting its applicability in clinical practice.^16^ The study hypothesized that patients with AS may experience changes in neck muscle size that are associated with pain, inflammation, and restricted movement. If this difference is detected, rehabilitation programs can be designed according to the affected muscle.

This study aimed to compare neck muscle thickness in individuals with AS and healthy participants using ultrasound, and to examine how these measurements relate to pain, disability, disease activity, quality of life, functional outcomes, and spinal mobility.

## Methods

### Study Design

The research was designed as a cross-sectional study with a case–control framework. The authors took into account all the recommendations described in the Declaration of Helsinki. Approval for the study protocol was obtained from the Ethics Committee of Hitit University (Protocol No: 2024-24, Date: June 5, 2024).

### Participants

This study was conducted at Erol Olçok Training and Research Hospital between June 2024 and December 2024. The AS group consisted of 30 patients aged 18-60 years who met the modified New York criteria for AS, while the control group included 30 healthy individuals. The control group consisted of participants without neck pain, with no history of cervical surgery, and without any medical conditions that could potentially contribute to neck symptoms. Physical activity level, occupational neck load, and subclinical postural characteristics were not specifically evaluated in this group. Individuals were excluded from the study if they: (1) had cervical disc herniation, (2) had a previous cervical trauma, (3) had undergone cervical surgery, (4) had a history of malignancy or systemic infection, (5) declined to provide informed consent, or (6) were pregnant.

### Clinical Assessment

For both the AS and control groups, demographic variables including age, sex, body mass index (BMI), and marital status were recorded. In the AS group, treatment status, duration since symptom onset, and time since initial diagnosis were documented separately. Neck range of motion (ROM) was assessed in 3 planes (flexion, extension, lateral flexions, rotations) with a double inclinometer. In the AS group, neck pain severity was assessed via the visual analog scale (VAS), neck pain-related disability level was assessed via the Neck Disability Index (NDI),^^17^^ quality of life was assessed via the Ankylosing Spondylitis Quality of Life questionnaire (ASQoL),^18^ disease activity was assessed via the Bath Ankylosing Spondylitis Activity Index (BASDAI),^19^ functional status was assessed via the Bath Ankylosing Spondylitis Functional Index (BASFI),^20^ and spinal mobility was assessed via the Bath Ankylosing Spondylitis Metrology Index (BASMI).^21^ Cervical mobility was further categorized based on BASMI cervical rotation cut-off values as normal (>70°), moderate (20-70°), and severe (<20°).

### Sample Size Calculation

A sample size estimation was performed using G*Power software (version 3.1.9.7, Düsseldorf, Germany). Based on an effect size of 0.77 derived from the findings of a previous study,^22^ and setting the alpha level at 0.05 with 80% statistical power, the analysis determined that each group required 28 participants.

### Ultrasound Measurement

All ultrasonographic images were acquired using real-time B-mode ultrasound equipment (Samsung RS85 Prestige, Medical Systems Corporation, South Korea) with a 5-12 MHz linear transducer by a radiologist with more than 10 years of experience in musculoskeletal ultrasound, who was blinded to the participants’ group allocation. All measurements were performed bilaterally with the muscles in a resting position. To improve standardization, all ultrasound measurements were performed using pre-warmed gel and minimal, consistent probe pressure. Once the optimal image was obtained, the image was frozen, and all measurements were performed on the frozen image. The ultrasound assessment of the neck muscles required approximately 10-15 minutes per participant, including patient positioning and image acquisition.

**Figure 1.**
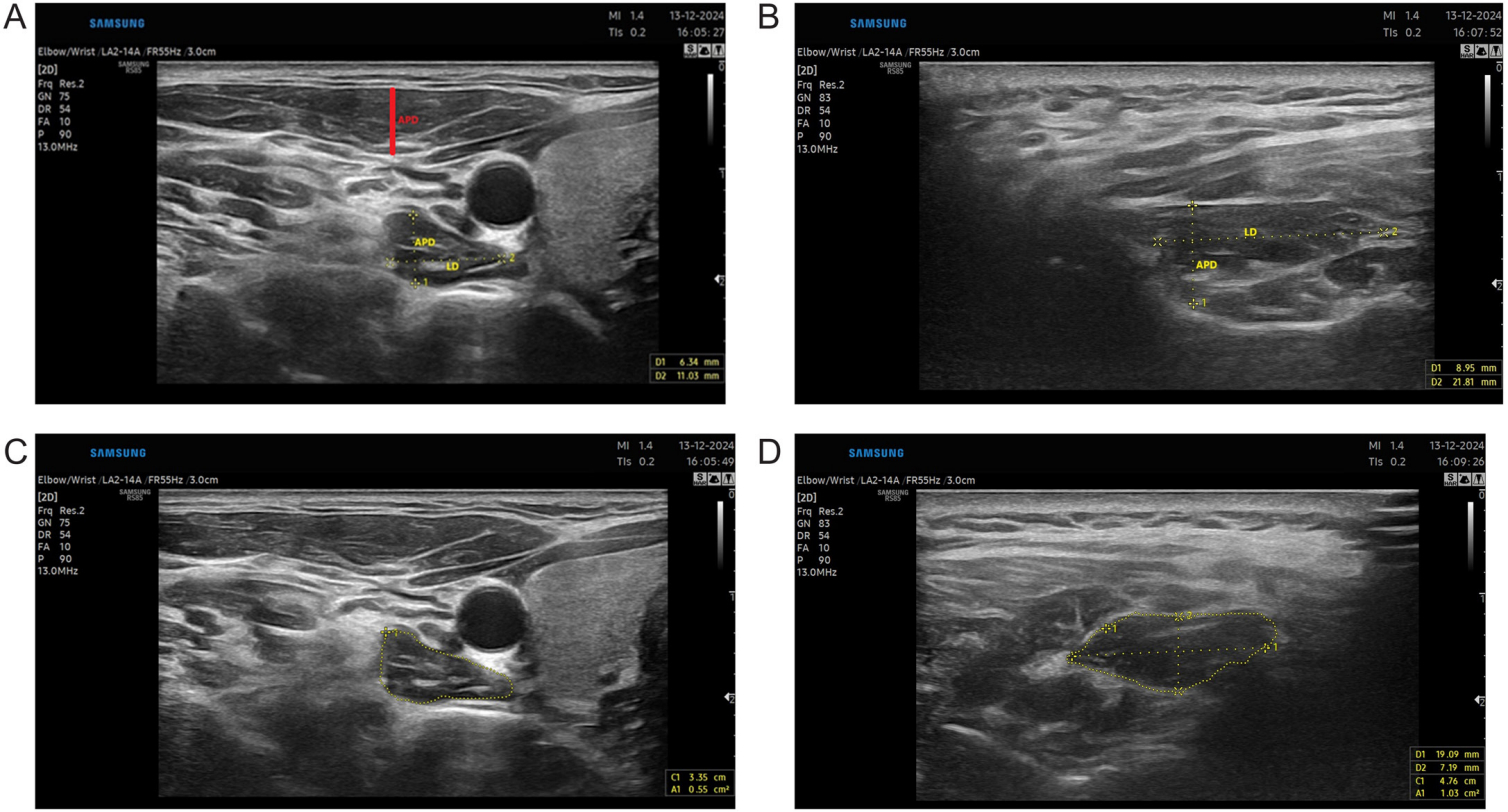
Ultrasound imaging of muscles. A) Dashed lines show the anteroposterior dimension (APD) and lateral dimension (LD) of the longus colli. The solid line shows the anteroposterior dimension of the sternocleidomastoid. B) Dashed lines show the anteroposterior dimension (APD) and lateral dimension (LD) of the cervical multifidus. C) Cross-sectional area measurement of the longus colli is shown. D) Cross-sectional area measurement of the cervical multifidus is shown.

For imaging of the LC muscle, participants were placed in the supine position with a small pillow supporting the head. The muscle was assessed at the C5-C6 level by positioning the transducer transversely approximately 2 cm inferior to the thyroid cartilage.^23^ Additionally, the thickest part of the SCM muscle was measured on this image (Figure 1A).

Visualization of the CM muscle was performed with participants seated and their head and neck maintained in a neutral alignment. The transducer was positioned transversely over the C4 spinous process and then shifted laterally to obtain the appropriate image. Once the vertebral lamina and fascia were clearly visible, the image was frozen and measurements were made^23^^,^^24^ (Figure 1B).

For both the CM and LC muscles, the lateral dimension was defined as the maximum horizontal distance between the medial and lateral margins. The anteroposterior dimension was obtained by drawing a perpendicular line from the midpoint of the lateral measurement, representing the greatest vertical depth from the ventral to the dorsal muscle borders.^3^ (Figure 1A and Figure 1B).

To measure the cross-sectional area (CSA) of the LC, the vertebral body in the medial and inferior regions, the carotid artery in the lateral region, and the retropharyngeal space in the superior region were considered boundaries^25^ (Figure 1C). The CSA of the CM was measured by taking the fascia between the CM and semispinalis superiorly, the spinous process medially, and the fascia between the CM and rotator muscles inferiorly as the border^26^ (Figure 1D).

### Statistical Analysis

Statistical analyses were performed using SPSS Statistics 21 (IBM SPSS Corp.; Armonk, NY, USA) and MedCalc version 23.3.2 (MedCalc Software Ltd.; Ostend, Belgium). Variables that followed a normal distribution were summarized as mean ± standard deviation, whereas non-normally distributed data were reported as median values. Categorical variables were compared using the chi-square test. For group comparisons, the independent Student’s *t*-test was applied to normally distributed variables, while the Mann–Whitney *U*-test was used for data that did not meet normality assumptions. In the AS group, treatment-related subgroup analyses were conducted using the Mann–Whitney *U*-test. Comparisons of neck muscle thicknesses across cervical mobility categories defined by BASMI cervical rotation cut-off values were performed using the Kruskal–Wallis test due to non-normal data distribution and the presence of more than 2 independent groups. Associations between neck muscle thickness and clinical parameters in the AS group were examined using partial correlation analysis, with adjustment for age and BMI. Multivariate linear regression analyses were conducted to examine the independent associations between muscle dimensions and clinical parameters, with adjustment for age and BMI. Considering the limited sample size and to preserve model stability, the number of predictors in each regression model was restricted to a maximum of 3. To mitigate multicollinearity resulting from bilateral measurements and strong intercorrelations among different muscle variables, only a single muscle parameter demonstrating the strongest statistically significant correlation coefficient (r) with the corresponding clinical outcome was included in each model. A 2-tailed *P* value of less than .05 was considered indicative of statistical significance

### Reliability

Intra-rater reliability was assessed by calculating the intraclass correlation coefficient. For this purpose, the same radiologist evaluated the neck muscles of 10 healthy volunteers twice at 5-day intervals via ultrasound. The intraclass correlation coefficients were 0.818 for the anteroposterior dimension of the LC, 0.875 for the lateral dimension of the LC, 0.936 for the CSA of the LC, 0.879 for the anteroposterior dimension of the CM, 0.891 for the lateral dimension of the CM, 0.866 for the CSA of the CM, and 0.815 for the anteroposterior dimension of the SCM. Intraclass correlation coefficient analysis revealed nearly perfect intra-rater reliability for all the measurements (0.80-1.00).

## Results

Thirty patients with AS (44.73 ± 1.56 years) and 30 healthy controls (41.43 ± 1.13 years) were evaluated. Both groups consisted of 12 women and 18 men. Patients in the AS group reported moderate neck pain, with a median VAS score of 5. The mean NDI score was 33.33 ± 18.03%, indicating a moderate level of neck-related disability. Sex, age, marital status, and BMI did not differ significantly between the AS and control groups. The participants’ demographic data are presented in Table 1.

The anteroposterior dimension and CSA of the right LC were significantly lower in the AS group than in the healthy group (mean difference (MD): 0.76, 95% CI: −1.41 to 0.10, *P* = .025, MD: 16.23 ,95% CI: −27.95 to 4.50, *P* = .021, respectively). Similarly, the anteroposterior dimension and CSA of the left LC were significantly lower in the AS group than in the control group (MD: 0.76, 95% CI: −1.40 to 0.11, *P* = .023, MD: 17.36, 95% CI: −28.78 to 5.93, *P* = .012, respectively). The lateral dimensions of both the right and left CMs were significantly lower in the AS group than in the control group (MD: 2.85, 95% CI: −4.44 to 1.25, *P* = .001, MD: 2.96, 95% CI: −4.76 to 1.15, *P* = .002, respectively). In contrast, no significant group differences were detected in the anteroposterior measurements of the SCM on either the right or left side (*P* = .460, *P* = .098, respectively) (Table 2).

In the AS group, subgroup analyses comparing patients receiving biologic therapy (n = 21) and those treated with only nonsteroidal anti-inflammatory drugs (n = 9) showed no significant differences in neck muscle thicknesses, clinical scale scores, and neck ROM (all *P* > .05).

The relationships between the bilateral LC thicknesses and other parameters in the AS group are given in Table 3. No significant relationships were found between CM and SCM thicknesses and these parameters. In the AS group, partial correlation analysis controlling for age and BMI revealed no significant associations between neck muscle thickness and symptom duration, disease duration, and neck pain intensity (VAS). A significant negative correlation was observed between the anteroposterior thickness of the right LC and the NDI, ASQoL, BASDAI, and BASFI scores. Similarly, the lateral dimension of the right LC showed significant negative associations with the NDI, ASQoL, BASDAI, and BASMI. Additionally, the CSA of the right LC demonstrated significant inverse correlations with the NDI, ASQoL, and BASMI (Table 3).

For the left LC, the anteroposterior measurement demonstrated significant negative correlations with NDI, ASQoL, BASDAI, and BASFI. Likewise, the lateral dimension of the left LC showed significant inverse associations with NDI, ASQoL, BASDAI, and BASMI. Furthermore, the CSA of the left LC was negatively correlated with NDI and ASQoL (Table 3).

According to BASMI cervical rotation cut-off values, 11 patients (36.7%) in the AS group were classified as having normal cervical mobility, 13 (43.3%) had moderate limitation, and 6 (20.0%) had severe limitation. Comparisons of neck muscle thicknesses across these categories revealed no significant differences between groups (all *P* > .05).

The relationships between right and left LC thicknesses and neck ROM in the AS group are given in Table 4. The lateral dimension of the right LC was positively correlated with neck extension (*r* = 0.375, *P* = .049), and the CSA of the right LC showed positive correlations with neck extension (*r* = 0.386, *P* = .042) and right lateral flexion (*r* = 0.384, *P* = .044). In addition, the lateral dimension of the left LC was positively correlated with neck extension (*r* = 0.387, *P* = .042). No statistically significant associations were observed between the thickness of the CM and SCM muscles and neck ROM.

The multivariate linear regression analyses are summarized in Table 5. The NDI model was statistically significant (*P* = .001) and accounted for 40% of the variance. The anteroposterior dimension of the left LC was independently and negatively associated with NDI scores (β = −0.69, *P* < .001), indicating that greater muscle thickness was associated with lower (better) disability levels. The ASQoL model showed marginal overall significance (*P* = .06); however, the anteroposterior dimension of the left LC remained significantly associated with ASQoL scores (β = −0.53, *P* = .009) within the model. Although the BASDAI model was not statistically significant overall (*P* = .16), the same LC parameter demonstrated a significant negative association with BASDAI scores (β = −0.44, *P* = .03) within the model. The BASFI model was statistically significant (*P* = .04), accounting for 17% of the variance, with the anteroposterior dimension of the right LC independently associated with functional impairment (β = −0.44, *P* = .02). Finally, the BASMI model was highly significant (*P* = .003), explaining 35% of the variance, and the lateral dimension of the left LC was independently associated with reduced spinal mobility (β = −0.33, *P* = .04).

## Discussion

In this study assessing neck muscle thickness in individuals with AS, both LC and CM muscle measurements were found to be reduced in patients compared with healthy controls. Additionally, in this patient group, LC thickness showed a negative correlation with NDI, ASQoL, BASDAI, BASFI, and BASMI, whereas a positive correlation was detected with neck extension and lateral flexion degrees. In multivariate regression analyses controlling for age and BMI, it showed that LC thickness was independently associated with disability (NDI), functional impairment (BASFI), and spinal mobility limitation (BASMI). Although the overall regression models for ASQoL and BASDAI did not reach statistical significance, LC thickness remained independently associated with these outcomes within the models. All significant coefficients were negative, indicating that lower LC thickness was associated with worse clinical outcomes in patients with AS.

The LC, one of the primary deep cervical flexor muscles, is essential for maintaining stability of the cervical spine.^27^ Therefore, the investigation of LC thickness and morphology in patients with neck pain has attracted scientific interest.^12^^,^^23^^,^^25^ Javanshir et al^^12^^ reported that individuals with chronic neck pain exhibited reduced anteroposterior thickness and CSA of the LC muscle compared with healthy participants, while lateral thickness did not differ between groups. They also identified a negative association between CSA and disability, as well as between anteroposterior thickness and pain intensity. Research involving patients with mechanical neck pain has further shown disrupted coordination between deep and superficial cervical flexors and diminished electromyographic activity in the deep flexor muscles. These findings support the idea that LC atrophy in chronic neck pain may be linked to motor control impairments and may adversely influence the dynamic function of the cervical spine. Likewise, research on individuals with cervical radicular pain has shown that the bilateral CSA of the LC muscle is reduced in these patients compared with healthy controls. However, it remains unclear whether LC atrophy in this patient group is the cause or consequence of pain.^25^ Avoiding movement due to pain may be associated with muscle atrophy, which can further exacerbate pain.^28^ Consistent with findings from studies on mechanical neck pain, the study found that both the anteroposterior thickness and CSA of the LC muscle were reduced in the AS group compared to healthy controls. LC atrophy may be associated with impaired cervical stabilization, reduced spinal mobility, increased pain, and poorer quality of life. One of the characteristic causes of AS is the development of cervical kyphosis.^29^ It is well known that deep flexor muscles weaken in cases of cervical kyphosis.^30^ In AS patients, LC muscle atrophy may be associated with cervical kyphosis. Considering the correlations between LC atrophy and disability, disease activity, quality of life, functional status, and spinal mobility, strengthening deep neck flexor muscles in this patient group may yield positive clinical outcomes.

The LC is a weak neck flexor, and Vasavada et al^^31^^ estimated that the longus muscles contribute approximately 17% of the total neck flexion moment. In the study, LC thickness showed selective associations with neck extension and lateral flexion rather than with flexion or rotation in the AS group. This pattern may reflect the role of the LC in maintaining cervical stability, supporting cervical lordosis, and contributing to postural control rather than gross movement production.

Studies evaluating the CM muscle in individuals with neck pain have yielded conflicting results.^8^^,^^22^ Among women with chronic neck pain, the CM muscle has been shown to have a reduced CSA relative to healthy individuals.^22^ Similarly, in patients with chronic unilateral cervical discopathy, a lower CSA of the CM was observed.^7^ In contrast, in chronic idiopathic neck pain, magnetic resonance imaging (MRI) revealed a larger CM muscle volume with increased fatty infiltration.^8^ A recent study also revealed greater fatty infiltration in the CM muscle in patients with cervical spondylosis.^32^ In a study conducted by Oztürk et al,^^33^^ AS patients presented greater fatty degeneration and denervation of the lumbar multifidus muscle than did nonradiographic patients with axial spondyloarthritis. This finding suggests that increased fatty infiltration in spinal muscles could contribute to greater pain severity and disability. In a study evaluating neck extensor muscles, similar to the study, the anteroposterior thickness of the CM was reportedly lower in the AS group than in the healthy control group.^24^ Morphological changes in the LC and CM may be associated with disuse, biomechanical compensation, or subclinical inflammation.

Falla et al^^34^^ demonstrated that, compared with healthy individuals, patients with neck pain show heightened activation of superficial cervical flexors and diminished recruitment of the deep flexor muscles. To evaluate this relationship, electromyographic measurements of the SCM, anterior scalene, and deep cervical flexor muscles were conducted during the craniocervical flexion test in women with chronic neck pain. The findings indicated that heightened activation of the superficial flexor muscles corresponded with reduced activity of the deep cervical flexors.^35^ Spinal kyphosis-induced forward head posture (FHP) is a major complication of AS.^36^ In FHP, which is associated with neck pain^37^ tightness and shortening are observed in the SCM and anterior scalene muscles, whereas elongation and weakness are observed in the deep neck flexor muscles.^38^ The literature presents conflicting evidence regarding cervical muscle thickness in patients with FHP.^39^^,^^40^ A study comparing asymptomatic women with and without FHP reported that SCM thickness was greater in those with FHP. This was attributed to tonic contraction, fatty infiltration, and underutilization of deep neck flexors.^40^ In research involving young adults with neck pain, those exhibiting FHP showed reduced SCM thickness compared with individuals with normal posture; however, this difference did not reach statistical significance.^39^ A recently published systematic review indicated that FHP is associated with increased SCM muscle thickness.^41^ In the study, however, this expected result was not obtained, and although it was not statistically significant, the SCM thickness was lower in the AS group. This finding may be associated with inflammation and immobilization, similar to what is observed in other neck muscles. Moreover, SCM thickness showed no significant correlation with clinical variables in patients with AS.

In the study, significant between-group differences were observed in the anteroposterior dimension and CSA of the LC muscle and in the lateral dimension of the CM, whereas no significant differences were detected in the remaining muscle measurements. In a recently published study, ultrasound demonstrated generally good to excellent intra-rater and test–retest reliability for anteroposterior, lateral, and CSA measurements of the LC, CM, and SCM muscles.^16^ However, several factors may influence measurement precision during the assessment of neck muscles, including small muscle size, deep location, and proximity to neurovascular structures.^16^ In addition, although skeletal muscles are 3-dimensional structures, the ultrasound probe used in this study provided 2-dimensional images. It has been shown that variations in probe orientation can affect the measurement of fascicle length and muscle fiber angles.^42^ Moreover, different muscle dimensions may exhibit varying sensitivity to structural changes. Taken together, these factors may partly explain the observed variability across different muscle dimensions in current findings.

In the present study, the majority of patients in the AS group were receiving biologic therapy. The absence of significant differences in muscle thicknesses and clinical outcomes in treatment-related subgroup analyses may be attributed to the small and imbalanced group sizes. A recent systematic review reported that biological disease-modifying anti-rheumatic drugs (bDMARDs) were associated with improvements in muscle strength and muscle mass in patients with spondyloarthritis and rheumatoid arthritis; however, no consistent effects on total lean body mass were demonstrated.^43^ Nevertheless, it remains unclear whether bDMARD therapy alone is sufficient to fully reverse muscle atrophy in spondyloarthritis.^44^

The literature indicates that lumbar paraspinal muscles have been studied more frequently than neck muscles in AS patients. In an MRI study evaluating lumbar paravertebral muscles in AS patients, the CSA of the lumbar muscles was similar in both groups, but AS patients exhibited greater fatty infiltration.^45^ Resorlu et al^^45^^ assessed the erector spinae and lumbar multifidus muscles in AS patients and healthy participants and reported that AS patients had a smaller muscle CSA and greater fatty infiltration.^46^ Lumbar paravertebral muscle atrophy in AS, believed to result from factors such as reduced mobility, chronic inflammation, and postural alteration, may also develop in the neck muscles. Associated with this atrophy, increased neck pain and disability, deterioration in quality of life and functional status, and limitations in neck movements may be observed. This limitation may further exacerbate muscle atrophy, creating a vicious cycle. Before this cycle occurs, it is considered beneficial to evaluate the cervical paravertebral muscles and design a personalized exercise program.

Exercise is a fundamental component of axial spondyloarthritis treatment and is recommended for all patients in the initial stage.^47^ A systematic review revealed that exercise had a moderate effect on function, disease activity, and spinal mobility in patients with AS.^^48^^ Owing to the methodological limitations of studies on exercise, no definitive conclusions can be reached as to which exercises are most appropriate. A recent study investigated the effectiveness of cervical stabilization exercises, particularly those designed to activate the deep neck muscles, in patients with axial spondyloarthritis. The group that performed the exercises showed improvement in the cervical position in all directions. It has been argued that this may be effective in improving impaired proprioception.^49^

In the present study, the ultrasonographic assessment of neck muscles was completed within approximately 10-15 minutes per participant and was performed by an experienced musculoskeletal radiologist, indicating that this method can be feasibly implemented in clinical practice when conducted by trained personnel. Based on these findings, ultrasound may represent a practical and objective tool for evaluating neck muscle involvement in patients with AS.

The study has several limitations. First, the lack of radiographic grading of cervical involvement and the absence of inflammatory markers may limit the ability to directly correlate muscle changes with the location and severity of the disease. Second, ultrasound measurements are rater dependent. The intra-rater reliability of the measurements was high; however, inter-rater reliability was not assessed, as another rater did not perform the ultrasound measurements. Third, muscle thickness was assessed without considering fatty infiltration. Since fatty infiltration can develop in AS patients without significant changes in muscle thickness,^45^ evaluating fatty infiltration in the examined muscles via MRI could be beneficial. Fourth, physical activity level, occupational neck load, and subclinical postural characteristics were not assessed in the control group. As these factors can influence neck muscle morphology, their potential confounding effects on the results cannot be excluded. Fifth, treatment-related subgroup analyses within the AS group may have been underpowered due to small and imbalanced subgroup sizes. Similarly, subgroup analyses based on cervical mobility categories did not reveal significant differences in neck muscle thicknesses, which may reflect limited statistical power rather than a true absence of group differences. Finally, due to the cross-sectional design of the study, causal relationships between neck muscle atrophy and clinical outcomes cannot be inferred. Future studies with larger, well-balanced cohorts and longitudinal designs are warranted to further clarify the relationships between treatment modalities, cervical mobility, neck muscle morphology, and clinical outcomes in AS.

The strengths of the study include that, to the best of knowledge, it is among the first studies to comprehensively evaluate LC, CM, and SCM muscle thickness using ultrasound in patients with AS and that the sample size was determined a priori by power analysis. Importantly, correlation and regression analyses were adjusted for age and BMI, strengthening the validity of the observed independent associations. These methodological considerations strengthen the rigor of the study and enhance the clinical relevance of the findings.

Compared with healthy individuals, patients with AS demonstrate a reduction in the thickness of the LC and CM muscles. Notably, reduced LC thickness was independently associated with greater disability, impaired functional status, and restricted spinal mobility. Although the overall regression models for BASDAI and ASQoL did not reach statistical significance, LC thickness remained independently associated with these outcomes, suggesting a potential relationship that warrants confirmation in larger cohorts.

Findings from the present study suggest that LC atrophy may be linked to poorer clinical outcomes in AS and underscore the importance of evaluating neck muscle morphology as part of comprehensive patient management.

Ultrasound is a valuable method for evaluating neck muscles in patients with AS. Future studies may investigate the role of ultrasound in planning rehabilitation programs and monitoring the effectiveness of the program according to the affected muscle in this patient group.

### Data Availability Statement:

The data that support the findings of this study are available on request from the corresponding author.

### Artificial Intelligence Usage Statement:

The authors declared that no Artificial Intelligence Tool was used in the preparation of the manuscript.

### Ethics Committee Approval:

This study was approved by Hitit University Faculty of Medicine Clinical Research Ethics Committee (Approval No: 2024-24, Date: June 5, 2024).

### Informed Consent:

Written informed consent was obtained from the patients who agreed to take part in the study.

### Peer-review:

Externally peer-reviewed.

### Author Contributions:

Concept – D.K., A.C.T.; Design – D.K., A.C.T.; Supervision – A.C.T.; Resources – D.K., O.K.; Materials – D.K., O.K.; Data Collection and/or Processing – D.K., O.K.; Analysis and/or Interpretation – D.K., O.K.; Literature Search – D.K., O.K.; Writing – D.K., O.K.; Critical Review – A.C.T.

### Declaration of Interests:

The authors have no conflicts of interest to declare.

### Funding:

The authors declare that this study received no financial support.

## Figures and Tables

**Table 1.** Demographic and Clinical Characteristics of the Participants

Characteristics	AS Group (n = 30)	Control Group (n = 30)	*P*
Age, years*	44.73 ± 1.56	41.43 ± 1.13	.092
Sex, F/M, n (%)	12/18 (40/60)	12/18 (40/60)	1.000
Marital status, single/married, n (%)	4/26 (13.3/86.7)	9/21 (30/70)	.117
BMI, kg/m^2^*	27.31 ± 4.19	25.56 ± 3.20	.075
Treatment, biologic therapy/NSAIDs only, n (%)	21/9 (30/70)	​	​
Symptom duration, years*	17.76 ± 9.23	​	​
Diagnosis duration, years*	10.60 ± 7.25	​	​
Neck flexion, degrees**	45 (0-45)	​	​
Neck extension, degrees**	37.5 (0-45)	​	​
Neck lateral flexion, right, degrees**	35 (0-45)	​	​
Neck lateral flexion, left, degrees**	35 (0-45)	​	​
Neck rotation, right, degrees**	52.5 (0-90)	​	​
Neck rotation, left, degrees**	50 (0-90)	​	​
Neck pain (VAS)**	5 (1-10)	​	​
NDI, %*	33.33±18.03	​	​
ASQoL*	8.63±5.66	​	​
BASDAI*	4.94±2.36	​	​
BASFI*	4.01±2.48	​	​
BASMI**	3.80 (1.40-7.0)	​	​
Cervical mobility category (BASMI cervical rotation), Normal (>70°)/Moderate (20-70°)/ Severe (<20°), n (%)	11/13/6 (36.7/43.3/20)	​	​

AS, ankylosing spondylitis; ASQoL, Ankylosing Spondylitis Quality of Life; BASDAI, Bath Ankylosing Spondylitis Activity Index; BASFI, Bath Ankylosing Spondylitis Functional Index; BASMI, Bath Ankylosing Spondylitis Metrology Index; BMI, body mass index; F, female; M, male; n, number of participants; NDI, Neck Disability Index, NDI presented as percentage score; VAS, Visual Analog Scale for Neck Pain Intensity.

^*^Mean ± standard deviation.

^**^Median (minimum-maximum).

**Table 2.** Comparison of Neck Muscle Thickness Between Groups

​Muscle	Mean ± SD		​Mean Difference	95% CI of the Difference		​*P*
	AS Group (n = 30)	Control Group (n = 30)		Lower	Upper	
Longus colli, right APD, (mm)	6.37 ± 1.50	7.13 ± 0.97	0.76	0.11	1.41	**.025**
Longus colli, right LD, (mm)	10.43 ± 2.92	11.07 ± 1.40	0.64	-0.54	1.82	.610
Longus colli, right CSA, (mm^2^)	58.50 ± 26.15	74.73 ± 18.58	16.23	4.51	27.95	**.021**
Longus colli, left APD, (mm)	6.42 ± 1.47	7.18 ± 0.97	0.76	0.11	1.40	**.023**
Longus colli, left LD, (mm)	10.37 ± 2.46	11.06 ± 1.41	0.69	-0.34	1.72	.574
Longus colli, left CSA, (mm^2^)	58.10 ± 24.57	75.46 ±19.35	17.36	5.93	28.78	**.012**
Cervical multifidus, right APD, (mm)	9.23 ± 2.43	8.53 ± 1.91	-0.70	-1.82	0.42	.209
Cervical multifidus right, LD, (mm)	19.07 ± 3.44	21.92 ± 2.68	2.85	1.25	4.44	**.001**
Cervical multifidus right CSA, (mm^2^)	158.56 ± 55.59	144.2 ± 39.86	-14.36	-39.35	10.63	.433
Cervical multifidus left, APD, (mm)	9.63 ± 2.95	8.56 ± 1.89	-1.07	-2.35	0.21	.212
Cervical multifidus left, LD, (mm)	18.94 ± 4.18	21.90 ± 2.61	2.96	1.15	4.76	**.002**
Cervical multifidus left CSA (mm^2^)	151.23 ± 45.73	143.86 ± 39.51	-7.37	-29.45	14.71	.584
SCM right, APD, (mm)	7.62 ± 1.26	8.03 ± 1.39	0.41	-0.27	1.09	.460
SCM left, APD, (mm)	7.57 ± 1.48	8.18 ± 1.40	0.61	-0.13	1.35	.098

Values of *P* < .05 were accepted as significant and are marked in bold. Values are presented as mean ± standard deviation. Mean difference was calculated as Control – AS.

AS, Ankylosing Spondylitis; n, number of participants; APD, Anteroposterior dimension; LD, Lateral dimension; CSA,Cross-sectional area; SCM, Sternocleidomastoid, CI, Confidence interval

**Table 3.** Correlations Between Longus Colli Thickness and Parameters In Ankylosing Spondylitis Group

​	Symptom Duration	Diagnosis Duration	Neck Pain (VAS)	NDI	ASQoL	BASDAI	BASFI	BASMI
Longus colli, right, APD	​	​	​	​	​	​	​	​
* r*	−0.136	0.171	−0.076	−0.628**	−0.453*	−0.392*	−0.417*	−0.126
* P*	.491	.384	.7	**<.01**	**.018**	**.015**	**.027**	.524
Longus colli, right, LD	​	​	​	​	​	​	​	​
* r*	−0.272	−0.051	−0.240	−0.581**	−0.434*	−0.390*	−0.369	−0.378*
* P*	.161	.796	.218	**<.01**	**.021**	**.04**	.053	**.04**
Longus colli, right, CSA	​	​	​	​	​	​	​	​
* r*	−0.273	−0.002	−0.118	−0.588**	−0.424*	−0.362	−0.362	−0.376*
* P*	.161	.992	.549	**<.01**	**.025**	.058	.059	**.049**
Longus colli, left, APD	​	​	​	​	​	​	​	​
* r*	−0.219*	0.112	−0.133	−0.648**	−0.482**	−0.405*	−0.404*	−0.119
* P*	.262	.571	.5	**<.01**	**<.01**	**.033**	**.033**	.546
Longus colli, left, LD	​	​	​	​	​	​	​	​
* r*	−0.280	−0.045	−0.267	−0.566**	−0.430*	−0.397*	−0.367	−0.388*
* P*	.148	.818	.17	**<.01**	**.022**	**.036**	.055	**.041**
Longus colli left, CSA	​	​	​	​	​	​	​	​
* r*	−0.309	−0.13	−0.188	−0.588**	−0.419*	−0.352	−0.354	−0.358
* P*	.11	.95	.339	**<.01**	**.026**	.066	.065	.061

Note: Values are partial correlation coefficients (r), adjusted for age and body mass index.Statistically significant correlations (*P* < .05) are highlighted in bold.

APD, anteroposterior dimension; ASQoL, Ankylosing Spondylitis Quality of Life; BASDAI, Bath Ankylosing Spondylitis Activity Index; BASFI, Bath Ankylosing Spondylitis Functional Index; BASMI, Bath Ankylosing Spondylitis Metrology Index; CSA, cross-sectional area; LD, lateral dimension; NDI, Neck Disability Index; VAS, Visual Analog Scale for Neck Pain Intensity

*P < .05.

**P < .01.

**Table 4.** Correlations Between Longus Colli Thickness and Neck Range Of Motion in Ankylosing Spondylitis Group

​	Neck Flexion	Neck Extension	Neck Lateral Flexion, Right	Neck Lateral Flexion, Left	Neck Rotation, Right	Neck Rotation, Left
Longus colli, right, APD	​	​	​	​	​	​
* r*	0.199	0.242	0.241	0.239	0.164	0.136
* P*	.310	.216	.216	.220	.405	.491
Longus colli, right, LD	​	​	​	​	​	​
* r*	0.309	0.375*	0.339	0.367	0.216	0.223
* P*	.109	**.049**	.078	.055	.270	.253
Longus colli, right, CSA	​	​	​	​	​	​
* r*	0.344	0.386*	0.361	0.384*	0.243	0.232
*P*	.073	**.042**	.059	**.044**	.212	.235
Longus colli, left, APD	​	​	​	​	​	​
* r*	0.201	0.251	0.23	0.224	0.123	0.094
* P*	.304	.197	.239	.251	.534	.634
Longus colli, left, LD	​	​	​	​	​	​
* r*	0.313	0.387*	0.337	0.36	0.242	0.257
* P*	.105	**.042**	.08	.06	.215	.186
Longus colli, left, CSA	​	​	​	​	​	​
* r*	0.314	0.368	0.32	0.334	0.223	0.217
* P*	.104	.054	.096	.082	.253	.266

Note: Values are partial correlation coefficients (r), adjusted for age and body mass index. Statistically significant correlations (*P* < .05) are highlighted in bold.

APD, anteroposterior dimension; CSA, cross-sectional area; LD, lateral dimension.

*P < .05;.

**P < .01.

**Table 5.** Multivariate Linear Regression Analysis Models for NDI, ASQoL, BASDAI, BASFI, and BASMI in Ankylosing Spondylitis Group

Outcome Measure	Predictor Variable	Model Sigificance (ANOVA F, *P*)	Adjusted R^2^	Standardized Coefficients (β)	*P*
NDI	Longus colli, left, APD	F (3.26) = 7.43, *P* = .001	0.40	−0.69	<.001
ASQoL	Longus colli, left, APD	F (3.26) = 2.71, *P* = .06	0.15	−0.53	.009
BASDAI	Longus colli, left, APD	F (3.26) = 1.84, *P* = .16	0.08	−0.44	.03
BASFI	Longus colli, right, APD	F (3.26) = 3.03, *P* = .04	0.17	−0.44	.02
BASMI	Longus colli, left, LD	F (3.26) = 6.21, *P* = .003	0.35	−0.33	.04

Note: The model was adjusted for age and body mass index.

APD, anteroposterior dimension; ASQoL, Ankylosing Spondylitis Quality of Life; BASDAI, Bath Ankylosing Spondylitis Activity Index; BASFI, Bath Ankylosing Spondylitis Functional Index; BASMI, Bath Ankylosing Spondylitis Metrology Index; LD, lateral dimension; NDI, Neck Disability Index
